# Phosphate solubilizing microorganisms: a sustainability strategy to improve urban ecosystems

**DOI:** 10.3389/fmicb.2023.1320853

**Published:** 2024-01-05

**Authors:** Yang Feng, Jing He, Hongchen Zhang, Xiaolin Jia, Youning Hu, Jianqing Ye, Xinyuan Gu, Xinping Zhang, Haoming Chen

**Affiliations:** ^1^School of Art and Design, Xijing University, Xi'an, China; ^2^Shaanxi Provincial Research Center of Public Scientific Quality Development and Cultural and Creative Industry Development, Xi'an, China; ^3^School of Biological and Environmental Engineering, Xi’an University, Xi'an, China; ^4^School of Art and Design, Xi’an University of Technology, Xi'an, China; ^5^School of Environmental and Biological Engineering, Nanjing University of Science and Technology, Nanjing, China

**Keywords:** phosphate, landscape ecosystem, urban environmental, plants, soil nutrients

## Abstract

Intensification of urban construction has gradually destroyed human habitat ecosystems. Plants, which serve as the foundation of ecosystems, require green, low-cost, and effective technologies to sustain their growth in stressful environments. A total of 286 keywords and 10 clusters from the bibliometric analysis of 529 articles (1999–2023) indicate the increasing importance of research on microbial functionality in landscape ecosystems. Phosphate solubilizing microorganisms (PSMs) also improve plant disease resistance, adaptability, and survival. PSMs are widely used to promote plant growth and improve ecological quality. They can increase the availability of phosphorus in the soil and reduce the dependence of plants on chemical fertilizers. Microorganisms regulate phosphorus as key tools in landscape ecosystems. Most importantly, in urban and rural landscape practices, PSMs can be applied to green spaces, residential landscapes, road greening, and nursery planting, which play significant roles in improving vegetation coverage, enhancing plant resistance, improving environmental quality, and mitigating the heat island effect. PSMs are also helpful in restoring the ecological environment and biodiversity of polluted areas, such as brownfields, to provide residents with a more liveable living environment. Therefore, the multiple efficacies of PSM are expected to play increasingly important roles in the construction of urban and rural landscape ecosystems.

## 1 Introduction

### 1.1 Plants play a key role in landscape ecosystems

As the building blocks of ecosystems, plants convert solar energy into chemical energy through photosynthesis and absorb water and nutrients from the soil to create organic matter that supports life (Barrios et al., 2018). Meanwhile, the roots, leaves, and other organs of plants can not only absorb water and nutrients but also convert carbon dioxide into oxygen and release it into the atmosphere to regulate the air quality of the entire environment (Raven, 2022). Plants have important physical and ecological functions in landscape ecosystems. For example, plant roots can hold soil particles and increase soil viscosity and stability to reduce soil and water loss and avoid natural disasters, such as landslides. Plants can also purify air by absorbing pollutants, capturing particles, and eliminating bacteria. Additionally, their roots can absorb nutrients and organic matter from the water, effectively mitigating water eutrophication and other environmental issues (Nowak et al., 2014; Bodnaruk et al., 2017). Additionally, plants provide habitats, food sources, and protection to maintain the diversity of plant and animal species within ecosystems (Maroyi, 2022). It should be emphasized that not only do the beautiful shapes and colors of plants have the ability to enhance emotions, but these natural esthetics can also have a positive impact on human health and well-being. As the basic components of landscape ecosystems, plants have a variety of interdependent ecological functions that play crucial roles in ecosystems. It is important for humans to make full use of plant resources to meet their own needs while simultaneously protecting and maintaining the ecological balance of ecosystems. Therefore, effective techniques (improvement, conservation, and restoration) are needed to ensure that plants continue to play an important and irreplaceable role in landscape ecosystems.

## 2 Microbial enhancement of P is a key mechanism for plant growth

### 2.1 Phosphorus is essential for plant growth and development

**Figure 1:**
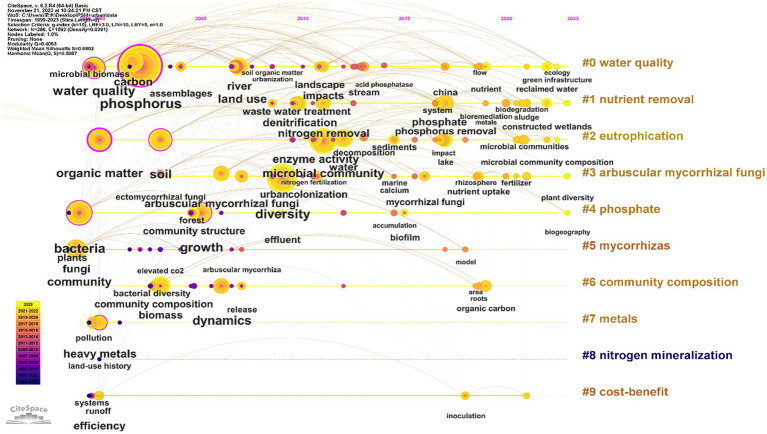
Keyword timeline clustering mapping based on CiteSpace.

As a component of many important substances and structures, such as nuclear proteins, phospholipids and nucleic acids, in plant cells, phosphorus plays an important role in physiological and biochemical reactions during the whole life cycle of plants and is indispensable for plant growth and development (Han et al., 2022). However, most phosphorus in the soil exists in the form of insoluble inorganic phosphorus or organophosphorus, which is difficult for plants to absorb and use (Wang et al., 2021). Although humans use phosphorus fertilizers to meet plant demand for phosphorus, the problems caused by long-term use have not been effectively solved, such as a decline in soil fertility, deterioration of physical and chemical properties, a decrease in microbial diversity, and heavy metal pollution (Vassilev et al., 2006; Kalayu, 2019), which not only threaten human production, life, health, and well-being, but also interfere with the sustainable development of landscape ecosystems. We conducted a bibliometric analysis of 529 articles on the topics of P, microbial, urban, and landscape [retrieved string: TS = (phosphorus) AND TS = (bacteria OR fungi OR microorganisms) AND TS = (urban OR landscapes)]. The presence of terms such as “phosphate” “decomposition” “nutrient uptake” “enzyme activity” and “organic carbon” indicates that functional studies on microbial mineralization and activation of insoluble phosphorus (#4 cluster) and interaction with plant roots (#1, #6, and #8 clusters) are gradually taking a dominant position in research (Figure 1).

### 2.2 PSMs are promoters of plant growth

In addition to protecting and maintaining plant resources, it is important to protect the microbial community within ecosystems. PSMs were initially discovered in agri-environmental research as a class of microorganisms that benefit plant growth (Vassilev et al., 2006). PSMs can convert insoluble phosphorus in the soil into P, which can be absorbed and utilized by plants by relying on organic acids, phosphatases, and other products through their own metabolism or synergistic effects with other organisms (Chen et al., 2019). Existing research has identified PSMs as an effective alternative to agrochemicals in flower cultivation, and it has shown a positive impact on the quality and production of flower crops, such as roses, marigolds, carnations, and jasmine (Zaidi et al., 2016). PSMs can also improve the plant absorption of nitrogen, potassium, calcium, and other mineral nutrients, thereby promoting plant growth and development (Ogut et al., 2011). Furthermore, PSMs not only supply phosphorus but also offer the auxins indole-3-acetic acid (IAA), gibberellic acid (GA), and cytokinin (CTK) to enhance plant root growth and development in soil, thereby increasing the water and nutrient absorption efficiency of plant roots. Additionally, they release antibiotics and produce B vitamins that suppress plant pathogens (Gupta and Kumar, 2017). In recent years, an increasing body of research has demonstrated the practical value of PSMs in enhancing plant landscape ecology. In landscape ecosystems, the addition of PSM-rich organic fertilizers and microbial agents to the soil not only improves soil properties but also reduces plant dependence on chemical fertilizers (Timofeeva et al., 2022; Sarmah and Sarma, 2023). Therefore, PSMs have broad prospects for application in plant cultivation and maintenance.

## 3 PSMs are an effective strategy to prevent future deterioration of landscape ecosystems

**Figure 2:**
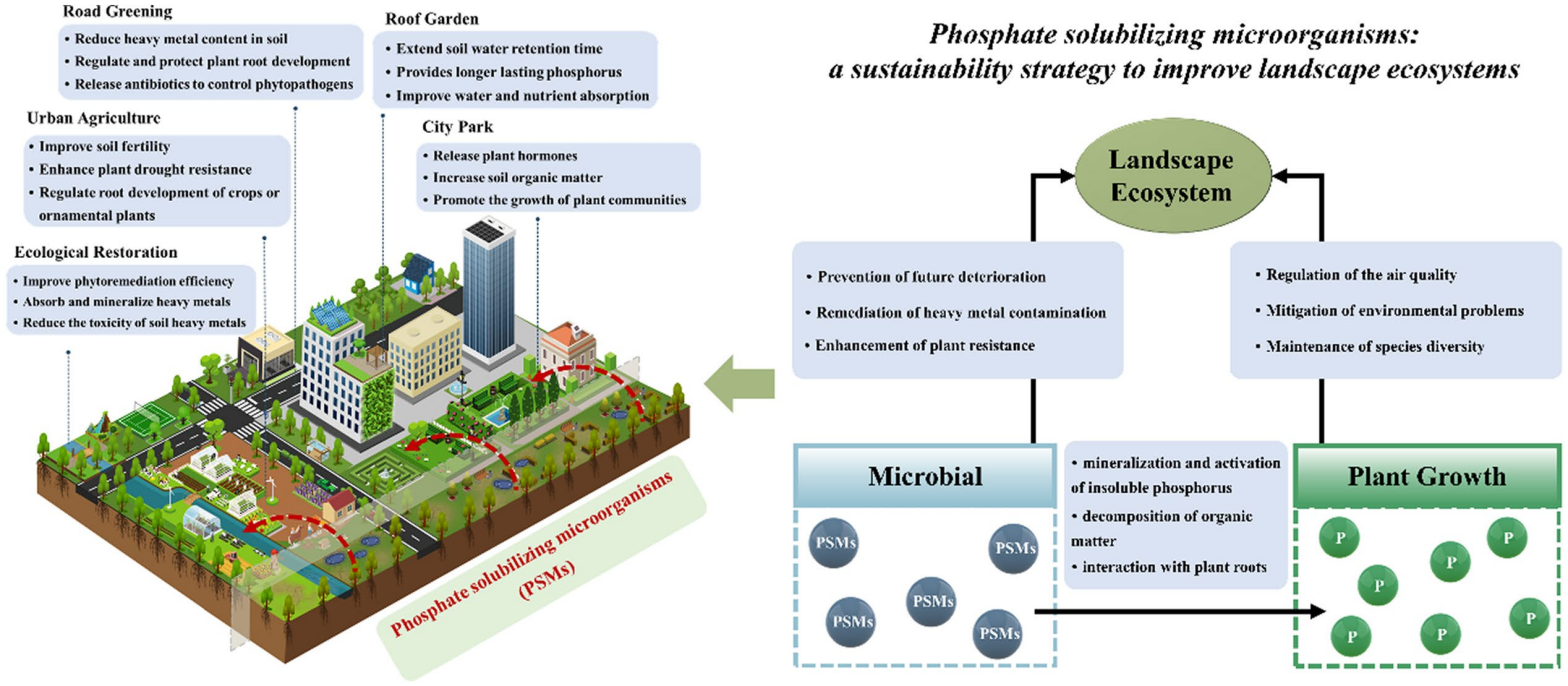
Schematic diagram of the mechanism by which phosphate-solubilizing microorganisms improve landscape ecosystem construction.

In urban and rural environments, the most serious problems in landscape ecosystems are the heat island effect, soil compaction, salinization, and poor plant diversity. These problems not only lead to steep increases in planting and maintenance costs but also increase the risk of imbalance in landscape ecosystems. PSMs have attracted widespread attention from the community precisely for their advantages in enhancing plant drought resistance, salt alkalinity resistance and insect resistance (Kalayu, 2019; Jin et al., 2022; Hu and Chen, 2023). In particular, PSMs can break down and remove pollutants and other harmful chemicals (e.g., pesticides, antibiotics, and toxic heavy metals), which also play a vital role in the health and stability of ecosystems (Figure 2).

### 3.1 PSMs can enhance plant resistance in urban and rural environments

Drought often leads to slow plant growth and development, aggravates desertification trends in landscape ecosystems, and seriously affects the balance of the ecological environment, the survival of ornamental plants, and agricultural production. Under drought stress, plants wilt with closed stomata and exhibit photosynthesis inhibition. PSMs can enhance plant activity and nutrient uptake capacity, and improve plant tolerance under drought stress (Khoshmanzar et al., 2020). Soil compaction and salinization severely inhibit the respiration and growth of plant roots, thereby slowing the growth and development of seedlings (Zhang et al., 2022). PSMs not only have strong saline-alkali tolerance but also enhance plant tolerance to saline-alkali stress (Jin et al., 2022). Studies have shown that PSMs can maintain the normal growth and metabolism of plants by controlling the production of osmotic adjustment substances and secondary metabolites (such as proline, soluble sugar, malondialdehyde, flavonoids and phenols) to balance the breakdown of reactive oxygen species in plants caused by drought, salinity and other stresses (Numan et al., 2018; Liang et al., 2019; Jin et al., 2022). Notably, some PSMs (*Aspergillus*, *Penicillium*, etc.) can eliminate the accumulation of reactive oxygen species by enhancing the activity of antioxidant enzymes in plants to reduce the damage caused by reactive oxygen species to plant cells (Li et al., 2017; Jin et al., 2022).

Most plants in urban and rural environments are artificially cultivated with a single plant diversity, serious diseases, and insect pests. Plant pests and diseases are primarily managed by chemicals. However, overuse of chemicals can lead to a range of environmental and social issues, including reduced plant yields, harm to animal ecosystems, and risks to human health. PSMs can improve the disease resistance of plants and reduce the damage caused by pathogenic bacteria to plants, with good biological control effects (Kalayu, 2019; Tian et al., 2021). For example, PSMs can resist the invasion of pathogenic bacteria by producing active metabolites (cyclic dipeptides, quinazolinones, and alcohols, etc.). Meanwhile, PSMs can improve plant resistance to pests and diseases by increasing the activities of plant disease resistance-related defense enzymes (Rawat et al., 2021; Palmieri et al., 2022; Tyśkiewicz et al., 2022).

### 3.2 PSMs can repair heavy metal pollution in urban and rural soil

Heavy metal residues in the soil not only affect plant growth but also pose a serious threat to the health of urban and rural habitats. Studies have shown that PSMs have a good fixation effect on heavy metal ions, such as Cr^6+^, Co^2+^, Cd^2+^, Zn^2+^, Cu^2+^, and Pb^2+^ in soil (Qian et al., 2019; Hu and Chen, 2023). PSMs can first dissolve insoluble phosphorus sources by releasing organic acids and enzymes and converting them to PO_4_^3−^ which binds to heavy metals to form phosphate precipitates and can greatly reduce the amount of free heavy metal ions in the soil (Chen et al., 2023). Second, PSM can secrete organic acids, indoleacetic acids, iron carriers, and other substances with oxidizing or reducing properties, thereby altering the metal valence state and diminishing the toxicity of toxic metals (Lai et al., 2022; Yu et al., 2022).

In conclusion, PSMs contribute significantly to landscape ecosystems. They not only directly promote plant growth, but also contribute to the remediation of existing environmental pollution and ecosystem deficiencies. This remediation process has a positive impact on the quality of human habitats and plays a crucial role in maintaining ecosystem stability and health.

## 4 Discussion and perspectives

Microbial fertilizers and preparations with specific functions are less expensive, greener, and more sustainable than chemical and physical amendments in the construction of urban green spaces and public spaces. Compared to other functional microorganisms, PSMs have more important application value and the potential to improve the ecology of plant landscapes because they can improve crop growth in several ways. These include the secretion of hormones favorable to plant growth, enhancement of soil fast-acting phosphorus, potassium, and other nutrients, improvement of soil pore structure, and reduction of pollution stress in the soil. However, there are several limitations to the use of PSM in highly polluted urban environments. The activity of microorganisms is affected by environmental factors, such as pH, temperature, humidity, and oxygen, which can limit their biological functions. In addition, owing to the wide variety of PSM species, the tolerance and remediation capacities of different types of PSM for contamination vary. Therefore, long-term microbial remediation requires a continuous investment of time and resources to maintain microbial activity and monitor the effectiveness of remediation. In addition, introducing PSMs to target sites and maintaining their activity is challenging because there is a need to address new ecological issues that may arise from microbial introduction. In summary, the benefits and limitations of PSM applications in landscape ecosystems coexist, and their combination with other remediation strategies and technologies for integrated management should be the focus of future research.

## Data availability statement

The original contributions presented in the study are included in the article/supplementary material, further inquiries can be directed to the corresponding authors.

## Author contributions

YF: Investigation, Methodology, Writing – original draft. JH: Methodology, Writing – original draft. HZ: Methodology, Writing – original draft. XJ: Conceptualization, Writing – original draft. YH: Data curation, Writing – review & editing. JY: Methodology, Writing – original draft. XG: Methodology, Writing – original draft. XZ: Visualization, Writing – review & editing. HC: Supervision, Validation, Writing – review & editing.
